# The silver(I) nitrate complex of the ligand *N*-(pyridin-2-ylmeth­yl)pyrazine-2-carboxamide: a metal–organic framework (MOF) structure

**DOI:** 10.1107/S2056989017003930

**Published:** 2017-03-21

**Authors:** Dilovan S. Cati, Helen Stoeckli-Evans

**Affiliations:** a Debiopharm International S.A., Chemin Messidor 5-7, CP 5911, CH-1002 Lausanne, Switzerland; b Institute of Physics, University of Neuchâtel, rue Emile-Argand 11, CH-2000 Neuchâtel, Switzerland

**Keywords:** crystal structure, metal-organic framework, MOF, silver(I), pyrazine, carboximide, pyridine, nitrate, hydrogen bonding

## Abstract

The reaction of silver(I) nitrate with the mono-substituted pyrazine carboxamide ligand, *N*-(pyridin-2-ylmeth­yl)pyrazine-2-carboxamide (**L**), led to the formation of the title compound with a metal–organic framework (MOF) structure, [Ag(C_11_H_10_N_4_O)(NO_3_)]_*n*_, poly[μ-nitrato-[μ-*N*-(pyridin-2-ylmethyl-κ*N*)pyrazine-2-carboxamide-κ*N* ^4^]silver(I)]. The silver(I) atom is coordinated by a pyrazine N atom, a pyridine N atom, and two O atoms of two symmetry-related nitrate anions. It has a fourfold N_2_O_2_ coordination sphere, which can be described as distorted trigonal–pyramidal. The ligands are bridged by the silver atoms forming **–Ag–L–Ag–L–** zigzag chains along the *a-*axis direction. The chains are arranged in pairs related by a twofold screw axis. They are linked *via* the nitrate anions, which bridge the silver(I) atoms in a μ_2_ fashion, forming the MOF structure. Within the framework there are N—H⋯O and C—H⋯O hydrogen bonds present.

## Abstract 2

The reaction of silver(I) nitrate with the mono-substituted pyrazine carboxamide ligand, *N*-(pyridin-2-ylmeth­yl)-pyrazine-2-carboxamide, led to the formation of a metal-organic framework (MOF) structure.

## Chemical context   

We have shown recently that by using silver(I) nitrate and various tetra­kis-substituted pyrazine ligands, one-, two- and three-dimensional coordination polymers can be formed (Assoumatine & Stoeckli-Evans, 2017 ▸). In the present report, the mono-substituted pyrazine carboxamide ligand, *N*-(pyridin-2-ylmeth­yl)pyrazine-2-carboxamide (**L**), whose crystal structure has been reported (Cati & Stoeckli-Evans, 2014 ▸), was reacted with silver(I) nitrate and led to the formation of a new compound with a metal–organic framework (MOF) structure, (I).
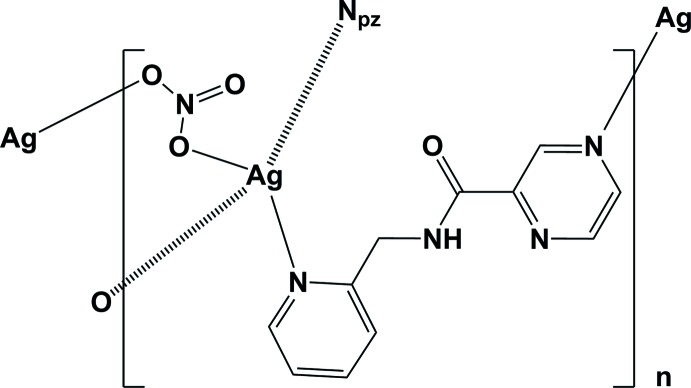


## Structural commentary   

**Figure 1:**
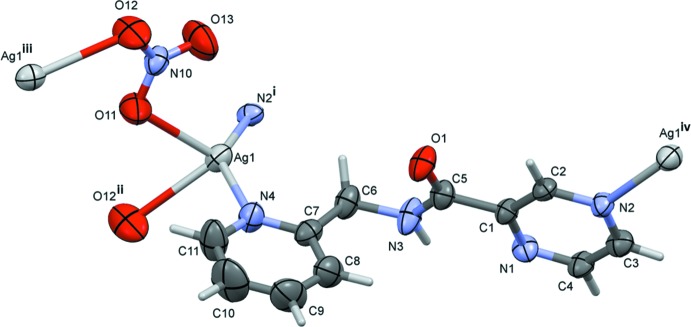
A view of the mol­ecular structure of the asymmetric unit of the title compound (I), with atom labelling. Displacement ellipsoids are drawn at the 50% probability level. For this figure, the symmetry codes are: (i) *x* − 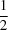, −*y*, *z*; (ii) −*x*, −*y* + 1, *z* + 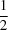; (iii) −*x*, −*y* + 1, *z* − 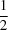; (iv) *x* + 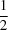, −*y*, *z*.

**Figure 2:**
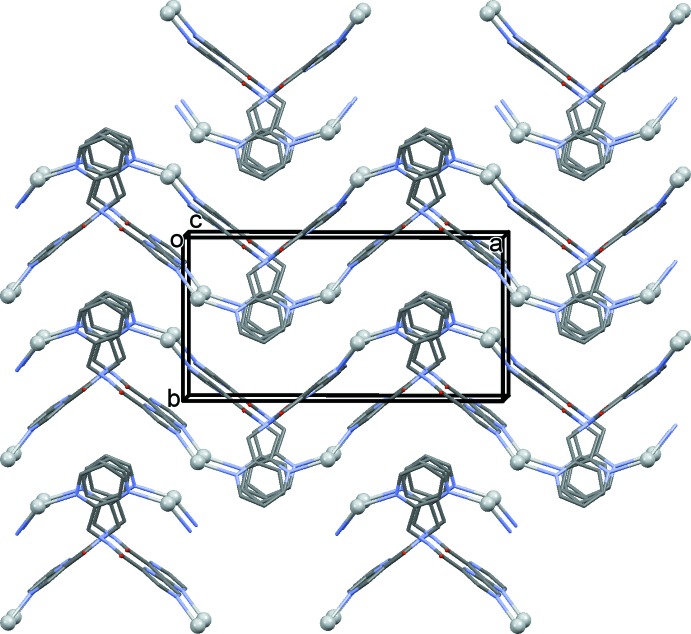
A view along the *c* axis of the **–Ag–L–Ag–L** zigzag chains propagating along the *a*-axis direction (silver atoms are grey balls and H atoms have been omitted for clarity).

The mol­ecular structure of the asymmetric unit of compound (I) is illustrated in Fig. 1 ▸. Selected bond lengths and angles involving the Ag1 atom are given in Table 1 ▸. Atom Ag1 is coordinated by a pyrazine N atom, N2, the pyridine N atom, N4, and two O atoms, O11 and O12, of two symmetry-related nitrate anions (Fig. 1 ▸ and Table 1 ▸). Therefore, atom Ag1 has a fourfold N_2_O_2_ coordination sphere and a distorted trigonal–pyramidal geometry with a τ_4_ parameter = 0.72 (τ_4_ = 1 for a perfect tetra­hedral geometry, 0 for a perfect square-planar geometry; for inter­mediate structures, including trigonal–pyramidal and seesaw, the values of τ_4_ fall within the range of 0 to 1.0; Yang *et al.*, 2007 ▸). Atom O13 of the nitrate anion lies above atom Ag1 with a distance Ag1⋯O13 of 2.864 (11) Å. The ligands are bridged by the silver atoms, forming **–Ag–L–Ag–L–** zigzag chains propagating along the *a*-axis direction (Fig. 2 ▸ and Table 1 ▸). They are arranged in pairs related by a twofold screw axis (Fig. 2 ▸).

## Supra­molecular features   

**Figure 3:**
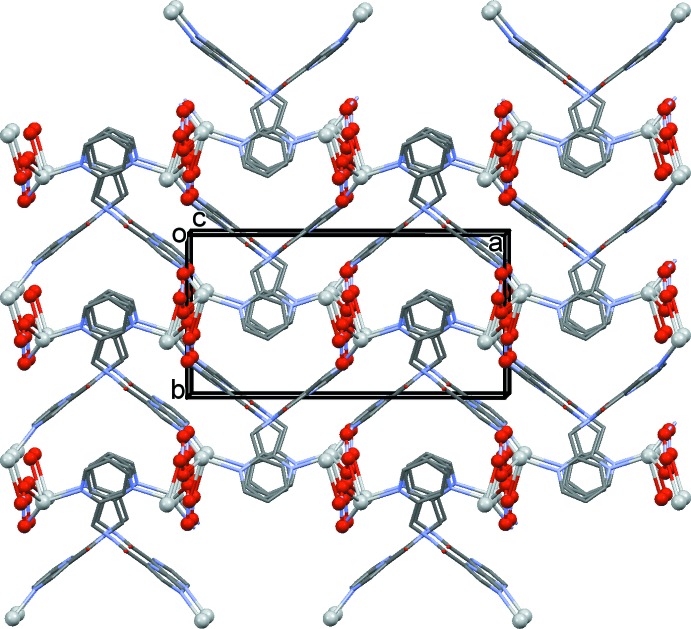
A view along the *c* axis of (I). The H atoms have been omitted for clarity, and the silver atoms and the nitrate anions are shown as balls.

**Figure 4:**
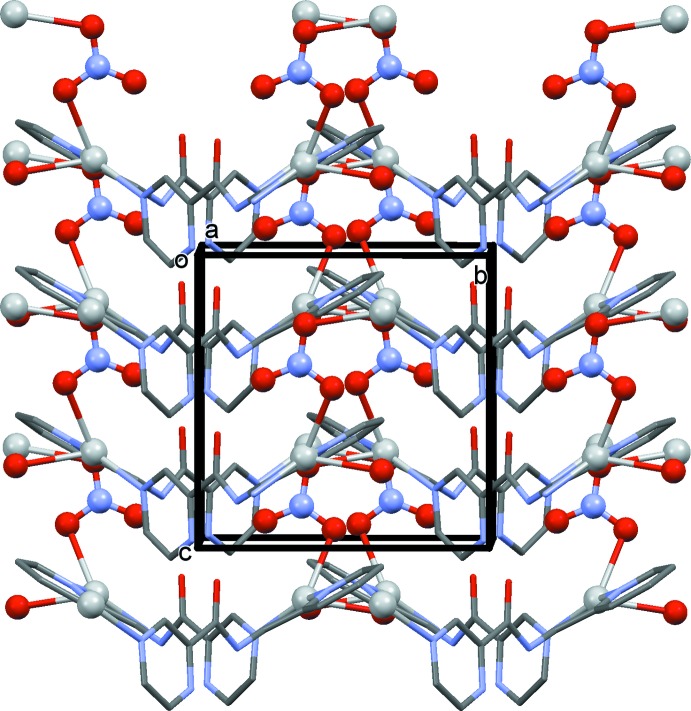
A view along the *a* axis of (I). The H atoms have been omitted for clarity, and the silver atoms and the nitrate anions are shown as balls.

In the crystal of (I), the chains are bridged by the nitrate anions, leading to the formation of the three-dimensional framework structure (Figs. 3 ▸ and 4 ▸). The nitrate anions bridge the silver atoms in a μ_2_ manner (Fig. 4 ▸), one of the many ways in which the nitrate anion inter­acts with silver atoms (Cambridge Structural Database; Groom *et al.*, 2016 ▸). Its role here is essential in forming the MOF structure.

Within the framework, there is an N—H⋯O hydrogen bond linking the amine group and carbonyl O atom of twofold-screw-related chains. There is also a C—H⋯O hydrogen bond present involving a pyrazine H atom and the third O atom of the nitrate anion, O13 (Table 2 ▸). There are small voids of *ca* 68 Å^3^ in the framework structure, equivalent to 4.8% of the volume of the unit cell.

## Database survey   

A search of the Cambridge Structural Database (Version 5.38, update February 2017; Groom *et al.*, 2016 ▸) for the title ligand (**L**) gave 15 hits. These include a report of the crystal structure of (**L**) (Cati & Stoeckli-Evans, 2014 ▸), and that of a silver(I) BF_4_^−^ coordination polymer (PORZOM; Hellyer *et al.*, 2009 ▸). Here the ligand bridges the silver(I) atoms, coordinating in a bidentate (*via* the pyridine N atom and the carbonyl O atom) and monodentate (to a pyrazine N atom) fashion, forming zigzag chains along [010]. The chains are linked by Ag⋯Ag contacts, of *ca* 3.32 Å, forming slabs (or metal–organic networks) lying parallel to the *bc* plane. The remainder of the hits in the above search are mainly first row transition metal complexes or coordination polymers.

## Synthesis and crystallization   

The synthesis of the ligand (**L**) has been described previously (Cati & Stoeckli-Evans, 2014 ▸). Ligand (**L**) (27 mg, 0.126 mmol) and AgNO_3_ (43 mg, 0.252 mmol) were introduced into 15 ml of aceto­nitrile in a two-necked flask (100 ml), isolated from the light by aluminium foil. The solution was refluxed for 5 h. The resulting limpid solution was filtered and the filtrate allowed to stand at room temperature. Colourless plate-like crystals were obtained in a few days (yield 42 mg, 87%).

Spectroscopic data: IR (KBr disc, cm^−1^): 3330 (*s*), 3063 (*m*), 1670 (*vs*), 1656 (*vs*), 1598 (*s*), 1571 (*s*), 1538 (*vs*), 1520 (*vs*), 1473 (*s*), 1463 (*s*), 1386 (*b* and *vs*), 1327 (*vs*), 1289 (*vs*), 1158 (*s*), 1101 (*m*), 1064 (*m*), 1023 (*s*), 877 (*w*), 825 (*m*), 776 (*m*), 706 (*m*), 667 (*s*), 611 (*m*), 533 (*m*), 456 (*m*). The broad and very strong absorption band at 1386 cm^−1^ indicates the presence of a coordinating nitrate anion. Elemental Analysis for AgC_11_H_10_N_5_O_4_ (*M*_r_ = 384.10 g mol^−1^): Calculated: C 34.40; H 2.62; N, 18.23%; found: C 34.58; H 2.55; N 18.05%.

## Refinement   

Crystal data, data collection and structure refinement details are summarized in Table 3 ▸. The NH H atom was located in a difference-Fourier map and freely refined. The C-bound H atoms were included in calculated positions and treated as riding: C—H = 0.94–0.98 Å with *U*_iso_(H) = 1.2*U*_eq_(C).

## Supplementary Material

Crystal structure: contains datablock(s) I, Global. DOI: 10.1107/S2056989017003930/zl2699sup1.cif

Structure factors: contains datablock(s) I. DOI: 10.1107/S2056989017003930/zl2699Isup2.hkl

CCDC reference: 1537331

Additional supporting information:  crystallographic information; 3D view; checkCIF report

## Figures and Tables

**Table 1:** Selected geometric parameters (Å, °)

Ag1—N2^i^	2.238 (7)	Ag1—O12^ii^	2.520 (9)
Ag1—N4	2.259 (8)	Ag1—O13	2.864 (8)
Ag1—O11	2.498 (9)		
			
N2^i^—Ag1—N4	140.8 (3)	N2^i^—Ag1—O12^ii^	115.0 (3)
N2^i^—Ag1—O11	117.1 (3)	N4—Ag1—O12^ii^	89.9 (4)
N4—Ag1—O11	98.5 (3)	O11—Ag1—O12^ii^	72.6 (3)

Symmetry codes: (i) 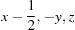; (ii) 
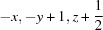
.

**Table 2:** Hydrogen-bond geometry (Å, °)

*D*—H⋯*A*	*D*—H	H⋯*A*	*D*⋯*A*	*D*—H⋯*A*
N3—H3*N*⋯O1^iii^	0.87 (3)	2.35 (12)	2.914 (12)	123 (11)
C2—H2⋯O13^iv^	0.94	2.59	3.330 (15)	136

Symmetry codes: (iii) 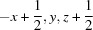; (iv) 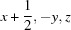.

**Table 3:** Experimental details

Crystal data	
Chemical formula	[Ag(C_11_H_10_N_4_O)(NO_3_)]
*M*_r_	384.11
Crystal system, space group	Orthorhombic, *P* *c* *a*2_1_
Temperature (K)	223
*a*, *b*, *c* (Å)	17.522 (3), 8.9559 (18), 8.9860 (13)
*V* (Å^3^)	1410.1 (4)
*Z*	4
Radiation type	Mo *K*α
μ (mm^−1^)	1.45
Crystal size (mm)	0.68 × 0.61 × 0.08

Data collection	
Diffractometer	STOE–Siemens AED2 four-circle
Absorption correction	Multi-scan (*MULABS*; Spek, 2009 ▸)
*T*_min_, *T*_max_	0.910, 1.000
No. of measured, independent and observed [*I* > 2σ(*I*)] reflections	3655, 2628, 2384
*R*_int_	0.022
(sin θ/λ)_max_ (Å^−1^)	0.605

Refinement	
*R*[*F* ^2^ > 2σ(*F* ^2^)], *wR*(*F* ^2^), *S*	0.047, 0.128, 1.10
No. of reflections	2628
No. of parameters	194
No. of restraints	2
H-atom treatment	H atoms treated by a mixture of independent and constrained refinement
Δρ_max_, Δρ_min_ (e Å^−3^)	1.04, −1.56
Absolute structure	Flack *x* determined using 1006 quotients [(*I* ^+^)−(*I* ^−^)]/[(*I* ^+^)+(*I* ^−^)] (Parsons *et al.*, 2013 ▸)
Absolute structure parameter	−0.06 (2)

Computer programs: *STADI4* and *X-RED* (Stoe & Cie, 1997 ▸), *SHELXS97* (Sheldrick, 2008 ▸), *SHELXL2014/6* (Sheldrick, 2015 ▸), *PLATON* (Spek, 2009 ▸), *Mercury* (Macrae *et al.*, 2008 ▸) and *publCIF* (Westrip, 2010 ▸).

## supplementary crystallographic information

### Crystal data

**Table:**

[Ag(C_11_H_10_N_4_O)(NO_3_)]	*D*_x_ = 1.809 Mg m^−^^3^
*M**_r_* = 384.11	Mo *K*α radiation, λ = 0.71073 Å
Orthorhombic, *P**c**a*2_1_	Cell parameters from 20 reflections
*a* = 17.522 (3) Å	θ = 10.0–13.4°
*b* = 8.9559 (18) Å	µ = 1.45 mm^−^^1^
*c* = 8.9860 (13) Å	*T* = 223 K
*V* = 1410.1 (4) Å^3^	Plate, colourles
*Z* = 4	0.68 × 0.61 × 0.08 mm
*F*(000) = 760	

### Data collection

**Table:**

STOE–Siemens AED2 four-circle diffractometer	2384 reflections with *I* > 2σ(*I*)
Radiation source: fine-focus sealed tube	*R*_int_ = 0.022
Plane graphite monochromator	θ_max_ = 25.5°, θ_min_ = 2.3°
ω/2θ scans	*h* = −21→21
Absorption correction: multi-scan (MULABS; Spek, 2009)	*k* = −10→10
*T*_min_ = 0.910, *T*_max_ = 1.000	*l* = −10→10
3655 measured reflections	2 standard reflections every 60 min
2628 independent reflections	intensity decay: 3%

### Refinement

**Table:**

Refinement on *F*^2^	Secondary atom site location: difference Fourier map
Least-squares matrix: full	Hydrogen site location: mixed
*R*[*F*^2^ > 2σ(*F*^2^)] = 0.047	H atoms treated by a mixture of independent and constrained refinement
*wR*(*F*^2^) = 0.128	*w* = 1/[σ^2^(*F*_o_^2^) + (0.0872*P*)^2^ + 0.889*P*] where *P* = (*F*_o_^2^ + 2*F*_c_^2^)/3
*S* = 1.10	(Δ/σ)_max_ < 0.001
2628 reflections	Δρ_max_ = 1.04 e Å^−^^3^
194 parameters	Δρ_min_ = −1.56 e Å^−^^3^
2 restraints	Absolute structure: Flack *x* determined using 1006 quotients [(*I*^+^)-(*I*^-^)]/[(*I*^+^)+(*I*^-^)] (Parsons *et al.*, 2013)
Primary atom site location: structure-invariant direct methods	Absolute structure parameter: −0.06 (2)

### Special details

**Table:**

Geometry. All esds (except the esd in the dihedral angle between two l.s. planes) are estimated using the full covariance matrix. The cell esds are taken into account individually in the estimation of esds in distances, angles and torsion angles; correlations between esds in cell parameters are only used when they are defined by crystal symmetry. An approximate (isotropic) treatment of cell esds is used for estimating esds involving l.s. planes.

### Fractional atomic coordinates and isotropic or equivalent isotropic displacement parameters (Å^2^)

**Table:**

	*x*	*y*	*z*	*U*_iso_*/*U*_eq_	
Ag1	0.05137 (3)	0.36456 (6)	0.69187 (15)	0.0402 (3)	
N1	0.3810 (5)	−0.0242 (8)	0.9863 (8)	0.0371 (16)	
N2	0.4836 (4)	−0.1928 (9)	0.8154 (8)	0.0338 (15)	
N3	0.2698 (5)	0.1329 (10)	0.8498 (9)	0.048 (2)	
H3N	0.279 (8)	0.131 (14)	0.945 (5)	0.08 (5)*	
N4	0.1730 (4)	0.4408 (9)	0.6583 (9)	0.045 (2)	
O1	0.3124 (5)	0.0642 (12)	0.6234 (8)	0.048 (2)	
C1	0.3775 (5)	−0.0301 (10)	0.8352 (9)	0.0322 (17)	
C2	0.4271 (6)	−0.1143 (9)	0.7518 (11)	0.0336 (18)	
H2	0.421457	−0.116833	0.647769	0.040*	
C3	0.4894 (6)	−0.1818 (12)	0.9640 (11)	0.039 (2)	
H3	0.529260	−0.232096	1.013043	0.047*	
C4	0.4380 (6)	−0.0979 (12)	1.0473 (10)	0.038 (2)	
H4	0.444189	−0.093686	1.151129	0.045*	
C5	0.3163 (7)	0.0593 (13)	0.7613 (12)	0.039 (3)	
C6	0.2071 (5)	0.2203 (12)	0.7917 (11)	0.044 (2)	
H6B	0.171943	0.242975	0.873408	0.053*	
H6A	0.179317	0.159729	0.718878	0.053*	
C7	0.2304 (5)	0.3641 (9)	0.7190 (11)	0.040 (3)	
C8	0.3053 (5)	0.4149 (12)	0.706 (2)	0.057 (3)	
H8	0.345961	0.359520	0.745841	0.068*	
C9	0.3187 (7)	0.5463 (16)	0.6332 (18)	0.074 (4)	
H9	0.368904	0.582281	0.625229	0.088*	
C10	0.2607 (11)	0.6256 (14)	0.573 (3)	0.086 (5)	
H10	0.269429	0.716443	0.523371	0.103*	
C11	0.1881 (7)	0.5672 (14)	0.5870 (18)	0.067 (3)	
H11	0.147229	0.619736	0.543869	0.081*	
N10	−0.0058 (5)	0.3498 (9)	0.3740 (9)	0.0390 (18)	
O11	−0.0039 (7)	0.4610 (9)	0.4543 (11)	0.072 (3)	
O12	−0.0112 (8)	0.3691 (9)	0.2369 (8)	0.074 (3)	
O13	−0.0028 (7)	0.2247 (10)	0.4254 (12)	0.085 (4)	

### Atomic displacement parameters (Å^2^)

**Table:**

	*U*^11^	*U*^22^	*U*^33^	*U*^12^	*U*^13^	*U*^23^
Ag1	0.0352 (4)	0.0473 (4)	0.0380 (4)	−0.0043 (2)	−0.0034 (4)	0.0055 (5)
N1	0.048 (4)	0.039 (4)	0.023 (3)	0.006 (3)	0.003 (3)	0.001 (3)
N2	0.036 (4)	0.037 (4)	0.028 (4)	0.006 (3)	−0.004 (3)	−0.001 (3)
N3	0.043 (5)	0.073 (6)	0.027 (4)	0.021 (4)	0.004 (4)	0.010 (4)
N4	0.037 (4)	0.046 (4)	0.051 (6)	0.001 (3)	−0.004 (3)	0.010 (4)
O1	0.047 (5)	0.074 (6)	0.024 (5)	0.017 (5)	0.001 (3)	0.006 (4)
C1	0.031 (4)	0.039 (4)	0.027 (4)	0.002 (3)	0.000 (3)	0.003 (3)
C2	0.037 (4)	0.036 (4)	0.028 (4)	0.000 (4)	−0.006 (4)	−0.001 (3)
C3	0.047 (6)	0.042 (5)	0.029 (5)	−0.004 (4)	−0.003 (4)	0.006 (4)
C4	0.043 (5)	0.047 (5)	0.024 (4)	0.002 (4)	−0.003 (3)	0.000 (4)
C5	0.040 (6)	0.043 (6)	0.034 (7)	0.003 (5)	0.001 (5)	0.007 (5)
C6	0.033 (5)	0.061 (6)	0.039 (5)	0.011 (4)	0.004 (4)	0.006 (4)
C7	0.036 (5)	0.049 (5)	0.035 (8)	0.000 (3)	−0.001 (4)	−0.004 (4)
C8	0.036 (4)	0.057 (5)	0.077 (8)	0.005 (4)	0.001 (7)	−0.011 (8)
C9	0.039 (6)	0.067 (8)	0.115 (13)	−0.007 (6)	0.004 (6)	−0.015 (7)
C10	0.079 (10)	0.055 (8)	0.125 (15)	−0.016 (7)	0.014 (10)	0.029 (8)
C11	0.051 (7)	0.053 (7)	0.098 (10)	0.001 (5)	0.005 (6)	0.031 (7)
N10	0.040 (4)	0.048 (5)	0.029 (4)	0.009 (3)	−0.001 (3)	0.002 (3)
O11	0.106 (7)	0.061 (6)	0.048 (4)	0.025 (6)	−0.024 (4)	−0.021 (5)
O12	0.135 (10)	0.064 (6)	0.022 (4)	0.005 (5)	0.012 (4)	0.004 (3)
O13	0.147 (11)	0.046 (5)	0.062 (6)	0.012 (6)	−0.008 (7)	0.011 (5)

### Geometric parameters (Å, º)

**Table:**

Ag1—N2^i^	2.238 (7)	C3—C4	1.392 (16)
Ag1—N4	2.259 (8)	C3—H3	0.9400
Ag1—O11	2.498 (9)	C4—H4	0.9400
Ag1—O12^ii^	2.520 (9)	C6—C7	1.500 (13)
Ag1—O13	2.864 (8)	C6—H6B	0.9800
N1—C4	1.317 (12)	C6—H6A	0.9800
N1—C1	1.360 (11)	C7—C8	1.395 (14)
N2—C2	1.342 (12)	C8—C9	1.36 (2)
N2—C3	1.343 (12)	C8—H8	0.9400
N3—C5	1.316 (14)	C9—C10	1.35 (2)
N3—C6	1.445 (12)	C9—H9	0.9400
N3—H3N	0.87 (3)	C10—C11	1.38 (2)
N4—C11	1.327 (14)	C10—H10	0.9400
N4—C7	1.334 (12)	C11—H11	0.9400
O1—C5	1.242 (10)	N10—O13	1.212 (12)
C1—C2	1.373 (13)	N10—O11	1.231 (12)
C1—C5	1.494 (14)	N10—O12	1.248 (11)
C2—H2	0.9400		
			
N2^i^—Ag1—N4	140.8 (3)	O1—C5—C1	120.1 (11)
N2^i^—Ag1—O11	117.1 (3)	N3—C5—C1	116.4 (9)
N4—Ag1—O11	98.5 (3)	N3—C6—C7	114.6 (8)
N2^i^—Ag1—O12^ii^	115.0 (3)	N3—C6—H6B	108.6
N4—Ag1—O12^ii^	89.9 (4)	C7—C6—H6B	108.6
O11—Ag1—O12^ii^	72.6 (3)	N3—C6—H6A	108.6
C4—N1—C1	115.5 (8)	C7—C6—H6A	108.6
C2—N2—C3	116.2 (8)	H6B—C6—H6A	107.6
C2—N2—Ag1^iii^	122.7 (6)	N4—C7—C8	120.4 (9)
C3—N2—Ag1^iii^	120.2 (7)	N4—C7—C6	114.6 (8)
C5—N3—C6	121.5 (9)	C8—C7—C6	125.0 (9)
C5—N3—H3N	118 (9)	C9—C8—C7	118.9 (11)
C6—N3—H3N	121 (9)	C9—C8—H8	120.5
C11—N4—C7	119.2 (9)	C7—C8—H8	120.5
C11—N4—Ag1	120.6 (7)	C10—C9—C8	121.0 (12)
C7—N4—Ag1	120.0 (6)	C10—C9—H9	119.5
N1—C1—C2	122.5 (8)	C8—C9—H9	119.5
N1—C1—C5	117.0 (8)	C9—C10—C11	117.2 (12)
C2—C1—C5	120.4 (8)	C9—C10—H10	121.4
N2—C2—C1	121.4 (9)	C11—C10—H10	121.4
N2—C2—H2	119.3	N4—C11—C10	123.4 (12)
C1—C2—H2	119.3	N4—C11—H11	118.3
N2—C3—C4	121.6 (10)	C10—C11—H11	118.3
N2—C3—H3	119.2	O13—N10—O11	121.5 (10)
C4—C3—H3	119.2	O13—N10—O12	120.5 (9)
N1—C4—C3	122.5 (9)	O11—N10—O12	118.0 (9)
N1—C4—H4	118.7	N10—O11—Ag1	103.4 (6)
C3—C4—H4	118.7	N10—O12—Ag1^iv^	108.1 (6)
O1—C5—N3	123.5 (12)		
			
C4—N1—C1—C2	3.7 (14)	C11—N4—C7—C8	−1.0 (16)
C4—N1—C1—C5	−176.6 (9)	Ag1—N4—C7—C8	−176.5 (9)
C3—N2—C2—C1	−1.3 (14)	C11—N4—C7—C6	−177.8 (11)
Ag1^iii^—N2—C2—C1	167.9 (6)	Ag1—N4—C7—C6	6.7 (11)
N1—C1—C2—N2	−1.7 (14)	N3—C6—C7—N4	176.6 (9)
C5—C1—C2—N2	178.5 (9)	N3—C6—C7—C8	−0.1 (15)
C2—N2—C3—C4	2.3 (15)	N4—C7—C8—C9	2 (2)
Ag1^iii^—N2—C3—C4	−167.2 (7)	C6—C7—C8—C9	178.3 (13)
C1—N1—C4—C3	−2.7 (14)	C7—C8—C9—C10	−1 (2)
N2—C3—C4—N1	−0.2 (16)	C8—C9—C10—C11	0 (3)
C6—N3—C5—O1	3 (2)	C7—N4—C11—C10	−1 (2)
C6—N3—C5—C1	−178.4 (9)	Ag1—N4—C11—C10	174.9 (14)
N1—C1—C5—O1	176.6 (13)	C9—C10—C11—N4	1 (3)
C2—C1—C5—O1	−3.7 (19)	O13—N10—O11—Ag1	19.1 (14)
N1—C1—C5—N3	−1.8 (15)	O12—N10—O11—Ag1	−160.9 (10)
C2—C1—C5—N3	178.0 (10)	O13—N10—O12—Ag1^iv^	164.7 (9)
C5—N3—C6—C7	−73.6 (14)	O11—N10—O12—Ag1^iv^	−15.2 (14)

Symmetry codes: (i) *x*−1/2, −*y*, *z*; (ii) −*x*, −*y*+1, *z*+1/2; (iii) *x*+1/2, −*y*, *z*; (iv) −*x*, −*y*+1, *z*−1/2.

### Hydrogen-bond geometry (Å, º)

**Table:**

*D*—H···*A*	*D*—H	H···*A*	*D*···*A*	*D*—H···*A*
N3—H3*N*···O1^v^	0.87 (3)	2.35 (12)	2.914 (12)	123 (11)
C2—H2···O13^iii^	0.94	2.59	3.330 (15)	136

Symmetry codes: (iii) *x*+1/2, −*y*, *z*; (v) −*x*+1/2, *y*, *z*+1/2.
